# A New Phase Change Material Based on Potassium Nitrate with Silica and Alumina Nanoparticles for Thermal Energy Storage

**DOI:** 10.1186/s11671-015-0984-2

**Published:** 2015-06-28

**Authors:** Manila Chieruzzi, Adio Miliozzi, Tommaso Crescenzi, Luigi Torre, José M Kenny

**Affiliations:** Civil and Environmental Engineering Department, University of Perugia, UdR INSTM, Strada di Pentima, 4-05100 Terni, Italy; ENEA – Italian National Agency for New Technologies, Energy and Sustainable Economic Development, Casaccia Research Centre, Via Anguillarese, 301-00123 S. Maria di Galeria, Rome Italy

**Keywords:** Phase change materials, Nanofluid, Thermal energy storage, Nanoparticles, Heat capacity, Molten salt, Nanocomposite

## Abstract

In this study different nanofluids with phase change behavior were developed by mixing a molten salt base fluid (KNO_3_ selected as phase change material) with nanoparticles using the direct synthesis method. The thermal properties of the nanofluids obtained were investigated. Following the improvement in the specific heat achieved, these nanofluids can be used in concentrating solar plants with a reduction of storage material. The nanoparticles used (1.0 wt.%) were silica (SiO_2_), alumina (Al_2_O_3_), and a mix of silica-alumina (SiO_2_-Al_2_O_3_) with an average diameter of 7, 13, and 2–200 nm respectively. Each nanofluid was prepared in water solution, sonicated, and evaporated. Measurements of the thermophysical properties were performed by DSC analysis, and the dispersion of the nanoparticles was analyzed by SEM microscopy. The results obtained show that the addition of 1.0 wt.% of nanoparticles to the base salt increases the specific heat of about 5–10 % in solid phase and of 6 % in liquid phase. In particular, this research shows that the addition of silica nanoparticles has significant potential for enhancing the thermal storage characteristics of KNO_3_. The phase-change temperature of potassium nitrate was lowered up to 3 °C, and the latent heat was increased to 12 % with the addition of silica nanoparticles. These results deviated from the predictions of theoretical simple mixing model used. The stored heat as a function of temperature was evaluated for the base salt, and the nanofluids and the maximum values obtained were 229, 234, 242, and 266 J/g respectively. The maximum total gain (16 %) due to the introduction of the nanoparticles (calculated as the ratio between the total stored heat of the nanofluids and the base salt in the range of temperatures 260–390 °C) was also recorded with the introduction of silica. SEM and EDX analysis showed the presence of aggregates in all nanofluids: with silica nanoparticles they were homogenously present while with alumina and silica-alumina also zones with pure salt could be detected.

## Background

Carbon dioxide is responsible for major man-made greenhouse effect, making it the most important contributor to climate change. The carbon dioxide emissions could still grow in the coming years as a result of the increased demand for world energy. In the absence of new policies, carbon dioxide emissions from the energy sector would increase by 61 % over 2011 levels by 2050 [1]. Policy choices and market developments that bring the share of fossil fuels in primary energy demand down to just under three quarters are not enough to stem the rise in energy-related carbon dioxide emissions, which grow by one fifth in 2040 [2].

To limit these emissions, it is necessary to make better use of the produced thermal energy by increasing the energy efficiency of industrial processes (heat recovery) and buildings and by increasing the use of renewable sources such as solar energy [3].

A key technological issue for solar thermal power plants and industrial waste heat recovery is to integrate an economic storage of thermal energy (Thermal Energy Storage–TES) [4–6], with the overall objective to increase the solar contribution, to improve efficiency, and to reduce the levelized energy cost (LEC).

Among the various methods of energy storage, latent heat thermal energy storage (LHTES) systems using phase change materials (PCMs) have been gaining importance in such fields as solar energy systems, district heating and cooling systems, energy-efficiency buildings [6–8] cool storage systems for central air-conditioning systems, and waste heat recovery systems [9]. This is mainly due to their high-energy storage density and their ability to provide heat at a constant temperature. Since the latent heat of fusion between the liquid and solid phases of PCMs is high compared to sensible heat, storage systems utilizing PCMs can be reduced in size respect to systems based on sensible heat. Therefore, several studies on PCM used as thermal energy storage material have been reported [9–14].

Phase change materials for thermal energy storage must have a large latent heat and a high thermal conductivity, a melting temperature in the practical range of operation, chemical stability, and must be low-cost, non-toxic, and non-corrosive. The most common PCMs studied during the last 40 years are mainly fatty acids, paraffin waxes, metal alloys, salts (fluorides, chlorides, hydroxides, carbonates, and nitrates) [15–18].

The addition of nanoparticles may induce an increase in both the thermal capacity and the thermal conductivity of the storage media [19–23]. This behavior along with the introduction of the concept of nanofluid, as a fluid with nanoparticles suspended by Brownian motion, was introduced in 1995 by Choi who showed an increase in thermal properties of some fluids when copper and aluminum nanoparticles were added [24]. The increase of the thermal capacity of a storage media may allow several advantages for the thermal energy storage systems since a high quantity of heat can be stored in a small volume of material. In this way the thermal storage systems become more compact, reducing the overall costs.

Research on nanofluids’ specific heat has been, however, limited compared to that on thermal conductivity [19–22, 25–27], although the evaluation of the heat capacity of these nanofluids is crucial since its improvement can reduce the amount of storage material.

In the past years several studies have been conducted on nanofluids based on mixtures of nitrates as PCM where KNO_3_ is one of the components [28–31]. In some of these studies, nanoparticles were also added [26, 32–36]. In any case no research was carried on to explore the enhancement of the specific heat capacity of nanofluids based on pure KNO_3_.

The aim of this work is then the development of a new phase change material with a melting temperature in the range 300–350 °C using different kinds of nanoparticles embedded in potassium nitrate as molten salt base material and the thermal characterization of the obtained nanofluid.

## Methods

### Materials

Potassium nitrate (KNO_3_) has been selected as PCM. It has a melting point of about 334 °C. This melting temperature is useful for many medium-low power applications. The salt was purchased from Sigma-Aldrich.

The selected nanoparticles were silica, alumina, and a hydrophilic fumed mixed oxide of silica and alumina. Table 1 shows the types of nanoparticle, the producers, and the nanoparticle sizes.

**Table 1:** Base salt and nanoparticles used for the nanofluids production

Material	Composition (wt. %)	Commercial name	Producer	Average diameter (nm)
KNO_3_	100	Potassium nitrate	Sigma-Aldrich (St. Louis, USA)	–
SiO_2_	100	Aerosil 300	Evonik (Germany)	7
Al_2_O_3_	100	Aeroxide Alu C	Evonik (Germany)	13
SiO_2_-Al_2_O_3_	82 ÷ 86 silica/14 ÷ 18 alumina	Aerosil Cok 84	Evonik (Germany)	2–200

The silica-alumina mixture was chosen since it was responsible of the higher enhancement of the specific heat for nanofluids based on potassium and sodium nitrate analyzed in our previous work [23]. The effect of the same mix of silica-alumina on potassium nitrate thus was worth investigating. Moreover, these nanoparticles show a wide range of dimension that could influence the thermal behavior of nanofluids.

The nanoparticle weight concentration of 1 % was chosen in this study taking into account that this is the percentage which gave good results in thermal properties enhancement as reported in literature for several nanofluids [22, 23, 25, 27].

### Experimental Procedures

The procedure followed to prepare the nanofluids consists of four steps [20]:

1. Amounts of 198 mg of potassium nitrate and 2 mg of nanoparticles were measured on a an analytical balance with ±0.1 mg precision (Mettler Toledo, type AB104-S, Greifensee, Switzerland) and put in a beaker.
2. Amount of 20 ml of distilled water was added to the potassium nitrate and the nanoparticles.
3. Potassium nitrate and the nanoparticles were dispersed in water using an ultrasonic bath for 100 min (ultrasonic bath, EMMEGI model AC-5).
4. The water solution was then kept at constant temperature (200 °C) on a hot plate to remove all the water for at least 2 h.

### Differential Scanning Calorimetry

The dry samples obtained were introduced in the differential scanning calorimeter (DSC) for thermal analysis by using a Mettler-Toledo DSC 822E/400. They were introduced in standard aluminum pans with lid and subjected to the following thermal cycle in nitrogen atmosphere: held at 250 °C for 5 min, heating from 250 to 390 °C at 20 °C/min, held at 390 °C for 5 min, cooling from 390 to 250 °C at 20 °C/min. Six consecutive cycles were run on each sample without opening the DSC furnace to ensure good mixing of the sample and reproducibility of the results. The DSC thermograms were analyzed and the phase-change heat, and melting temperatures were obtained using the software STARe. Moreover, the calorimetric data were used to calculate the specific heat (Cp) of the samples.

The pure salt was also tested in the same manner and the melting properties compared with those obtained with the nanofluids.

The measurements were also compared with a theoretical model (“simple mixing model”) based on the assumption of thermal equilibrium between the particles and the surrounding fluid [22, 37]:

1 $$ {C}_{\mathrm{p},\mathrm{n}\mathrm{f}}=\frac{\rho_{\mathrm{np}}{\phi}_{\mathrm{np}}{C}_{\mathrm{p},\mathrm{n}\mathrm{p}}+{\rho}_{\mathrm{f}}{\phi}_{\mathrm{f}}{C}_{\mathrm{p},\mathrm{f}}}{\rho_{\mathrm{np}}{\phi}_{\mathrm{np}}+{\rho}_{\mathrm{f}}{\phi}_{\mathrm{f}}} $$

where *C*_p_ is specific heat, *ϕ* is the volume fraction, *ρ* is the density, and the subscripts np, nf, and f refers to nanoparticle, nanofluid, and base fluid, respectively.

### Microstructural Characterization

The dispersion of nanoparticles was analyzed with a field emission scanning electron microscope (FESEM model SUPRA25, ZEISS, Oberkochen, Germany). The test was performed on one specimen for each system after DSC measurement. Each sample was metallized with a thin layer of gold (15 nm, 99.99 % of gold, 2 × 10^−6^ Torr) in a thermal evaporator (Sistec thin film equipment model GP 20 by Kenosistec Angelantoni Group, Massa Martana (PG), Italy). For the surface analysis secondary electrons were used.

Energy-dispersive X-ray (EDX) analysis (INCA, Oxford Instruments, UK) was also performed on the nanofluids with the same FESEM.

## Results and Discussion

### Heat of Fusion of Nanofluids

**Fig. 1:**
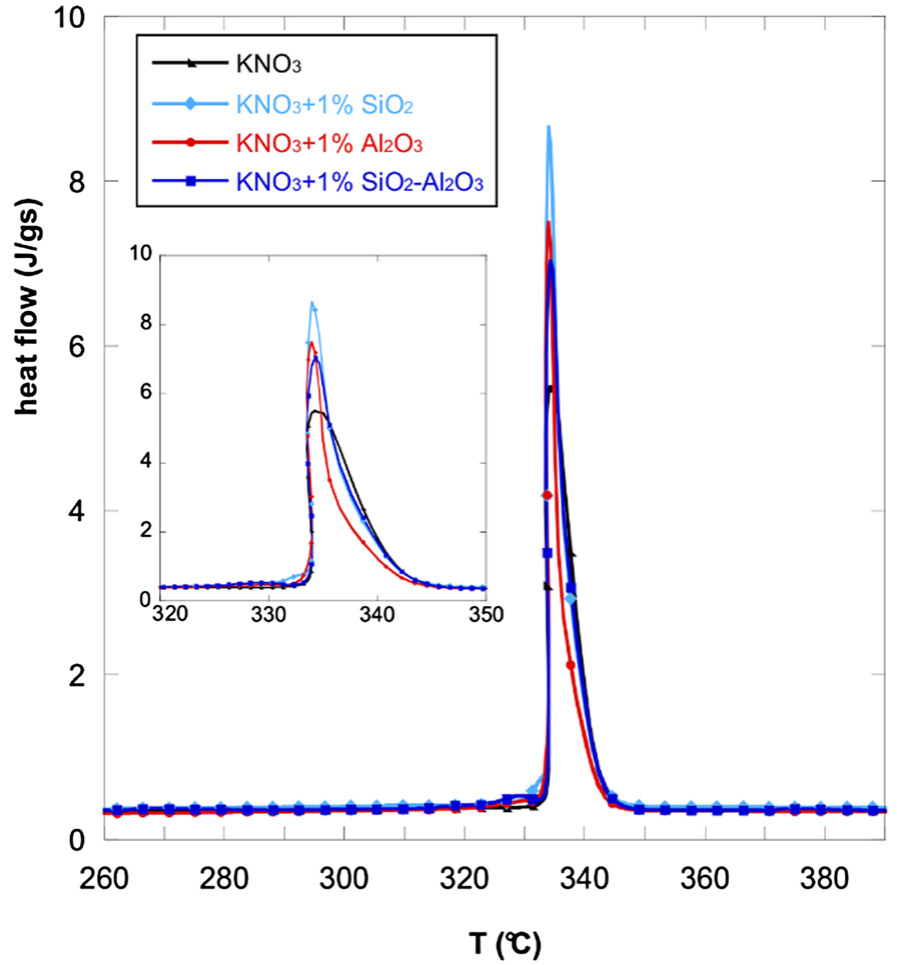
Heat flow curves. Heat flow versus temperature for KNO_3_ and the nanofluids with 1.0 wt.% of oxide nanoparticles

Figure 1 shows the heat capacity of KNO_3_ and the nanofluids. The analyzed salt and the nanofluids obtained with the addition of nanoparticles show a range of melting temperatures. The most significant temperature was taken as the onset temperature. Above this temperature the salt and the nanofluids start to melt.

Moreover, the different nanoparticles induce a change in the shape of the heat flow curve of the base salt that consequently modifies the values of the heat of fusion and the melting point of the nanofluids. These values were obtained by analyzing the DSC data and they are listed in Table 2.

**Table 2:** Heat of fusion, onset temperature of KNO_3_, and the nanofluids obtained with 1.0 wt.% of different nanoparticles

Material	Heat of fusion (J/g)	Onset temperature (°C)
KNO_3_	91.61	335.7
KNO_3_ + SiO_2_	102.46	333.7
KNO_3_ + Al_2_O_3_	92.10	333.3
KNO_3_ + SiO_2_-Al_2_O_3_	82.90	333.9

The latent heat of the pure KNO_3_ was compared to the literature data so the DSC measurement reliability was verified. In particular, the heat of fusion found in this work (91.61 J/g) is close the values reported in literature [14, 38–41].

As it can be seen, the addition of 1.0 wt.% of nanoparticles leads to a little decrease of onset temperatures when silica, alumina, or a mix of the two is used. In particular, with the addition of SiO_2_ nanoparticles, the phase-change temperature of potassium nitrate was lowered by about 2–3 °C.

The latent heat was also affected by the addition of nanoparticles. In particular, the addition of 1.0 wt.% of SiO_2_ and the mix of SiO_2_ and Al_2_O_3_ nanoparticles produced an enhancement of the heat of fusion for the nanofluids. The higher enhancement was obtained with SiO_2_ (12 %). The effect of such enhancement is more effective heat storage per unit volume.

The scanning rate adopted is higher than the conventional used for latent heat evaluation. However, in a recent work [35], a heating rate of 40 °C/min was used to minimize the precipitation of nanoparticles during DSC measurement.

In any case lower scanning rates (more suitable for latent heat measurements) were used and the results were still considered accurate. For these reasons, the rate of 20 °C/min was chosen in order to compare our results (especially the specific heat evaluation) to the scientific literature data on nanofluids, considering this choice as a good compromise between the optimal scanning rate for latent heat and specific heat evaluation.

### Heat Capacity of Nanofluids

**Fig. 2:**
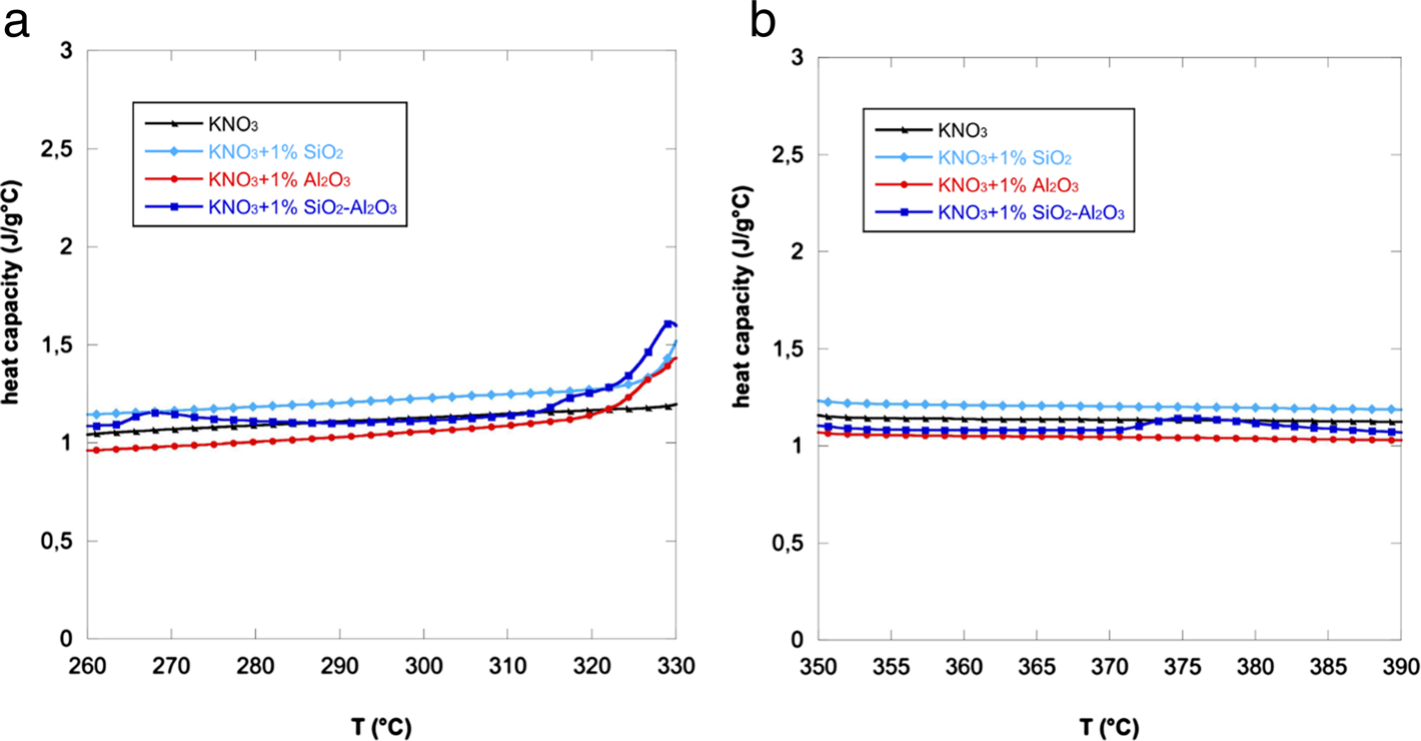
Specific heat curves. Variation of the specific heat with temperature of KNO_3_ and the nanofluids in the solid phase (**a**) and in the liquid phase (**b**)

The addition of different nanoparticles produced also an effect on the heat capacity of potassium nitrate both in solid and liquid phases. The curves related to the specific heats of the nanofluids versus temperature are reported in Fig. 2a (in the solid phase) and in Fig. 2b (in the liquid phase). The potassium nitrate is also reported in the same figure for comparison.

The specific heat of the pure KNO_3_ was also close to literature data [39] that reports specific heats in the range of temperature of solid state (260–390 °C) of 1.29–1.43 J/g °C and of 1.38–1.39 in the liquid state (in accordance with other works [40, 42]) and slightly lower with respect to the previous references [43, 44].

As it can be seen, the specific heat of the nanofluids generally increases with temperature between 260 and 330 °C (i.e., solid phase) while it remains constant at higher temperatures (i.e., liquid phase) [15].

Figure 2 demonstrates that the addition of silica nanoparticles significantly increases the specific heat of the base salt both at low and high temperatures. On the other hand, alumina seems not efficient in enhancing the heat capacity of potassium nitrate since its curve is always below KNO_3_ curve.

The specific heats were also calculated as average of single values recorded in the temperature range 260–330 °C for the solid phase and in the range 350–390 °C for the liquid phase. All the values (representing the average of three measurements) are reported in Table 3, which shows the effect of the nanoparticle addition on the heat capacity of potassium nitrate.

**Table 3:** Specific heat of KNO_3_ and the nanofluids obtained with 1.0 wt.% of different nanoparticles

	KNO_3_		KNO_3_ + SiO_2_		KNO_3_ + Al_2_O_3_		KNO_3_ + SiO_2_-Al_2_O_3_	
	*C*_p_ (J/g °C)		*C*_p_ (J/g °C)		*C*_p_ (J/g °C)		*C*_p_ (J/g °C)	
	Solid	Liquid	Solid	Liquid	Solid	Liquid	Solid	Liquid
First run	1.169	1.196	1.294	1.281	0.996	1.123	1.369	1.386
Second run	1.197	1.205	1.298	1.286	1.194	1.185	1.385	1.390
Third run	1.199	1.210	1.307	1.287	1.206	1.182	1.396	1.363
Fourth run	1.173	1.198	1.290	1.267	1.150	1.114	1.347	1.306
Fifth run	1.121	1.136	1.263	1.240	1.120	1.082	1.283	1.256
Sixth run	1.118	1.134	1.224	1.203	1.068	1.043	1.171	1.095
Enhancement (%)	–	–	9.5	6.1	−4.5	−7.8	4.7	−3.4

The average specific heat of the base salt in the liquid phase is 1.118 J/g °C. The higher increase of *C*_p_ was obtained with silica nanoparticles–9.5 % in solid phase and 6.1 % in liquid phase.

**Fig. 3:**
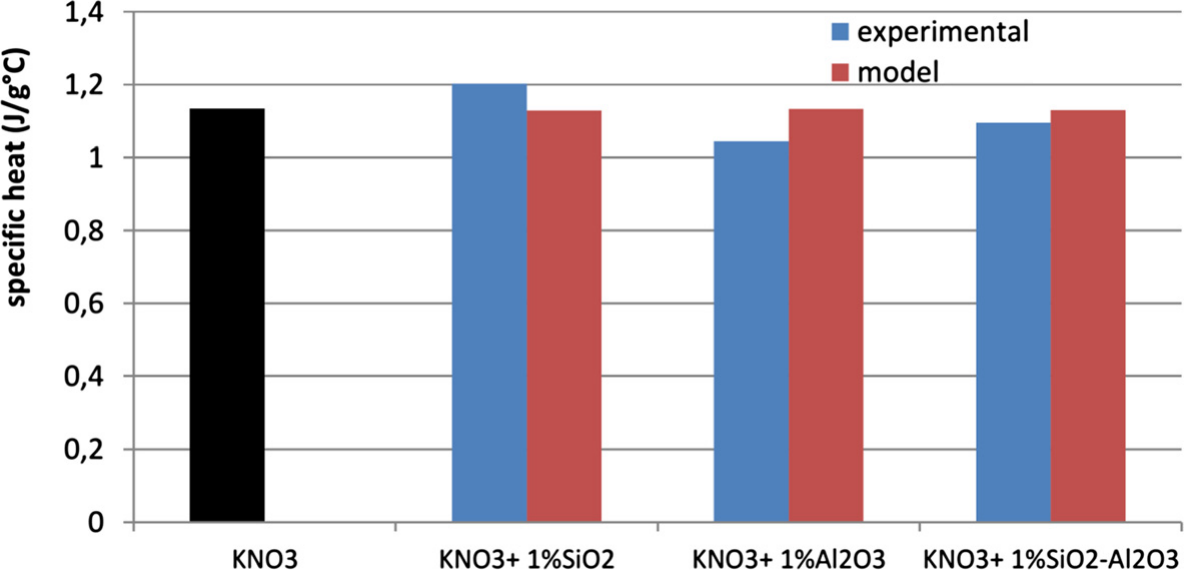
Experimental and theoretical specific heat values. Comparison between KNO_3_ and the nanofluids with 1.0 wt.% of oxide nanoparticles

The theoretical prediction of specific heats using Eq. (1) for the nanofluids with 1.0 wt.% of SiO_2_, Al_2_O_3_, and SiO_2_-Al_2_O_3_ nanoparticles is reported in Fig. 3 along with the experimental values of each nanofluid.

The nanoparticle density and *C*_p_ values used in the model were 2650, 3970, and 2860 kg/m^3^ and 0.70, 1.10, and 0.80 J/g °C for SiO_2_, Al_2_O_3_, and SiO_2_-Al_2_O_3_, respectively. For the potassium nitrate a density value of 2109 kg/m^3^ was used and the experimental *C*_p_ value reported in Table 3 (sixth run). As it can be seen, the theoretical values calculated with Eq. (1) for all nanofluids are lower than the specific heat of the neat potassium nitrate. This reduction is mainly due to the low specific heat of oxide nanoparticles with respect to the base salt [20–22].

Moreover, only the experimental specific heat value of the nanofluids with 1.0 wt.% of silica nanoparticles resulted higher than the value predicted by the model and higher than the base salt. This different behavior was explained by admitting the existence of other mechanisms for nanofluids [20].

The enhancement of specific heat could be associated to the highly specific surface energies related to the high surface area of the nanoparticles per unit volume [45, 46]. The nanoparticle diameter can play an important role since smaller nanoparticles give a greater solid/liquid interface area, thus increasing the contribution of interfacial effects in the corresponding suspensions [47, 48]. The solid–liquid interface may change the phonon vibration mode near the surface area of a nanoparticle and thus change the specific heat capacity of a nanofluid [22]. The highly specific interfacial area of the nanoparticle can adsorb liquid molecules to its surface forming liquid layers. These layers would constrain the nanoparticles and modify its free-boundary surface atoms into non-free interior atoms [46]. The change in the specific heat capacity of nanofluid is then due to the varied Gibbs free energy of nanoparticle and liquid layers.

### Heat Storage of Nanofluids

In order to understand the real effect of the nanoparticles on the storage capacity of the material, the data obtained from DSC tests were elaborated and the stored heat as a function of the temperature was evaluated. The storage capacity of each nanofluid (Eq. 2) is obtained from the integration of the heat flow curve in the range of temperatures between the minimum working temperature related to the solid phase (260 °C) and the maximum working temperature related to the liquid phase (390 °C):

2 $$ \mathrm{Stored}\_\mathrm{heat}\left(T \min, T \max \right)={\displaystyle \underset{T \min }{\overset{T \max }{\int }}h(T)dT} $$

**Fig. 4:**
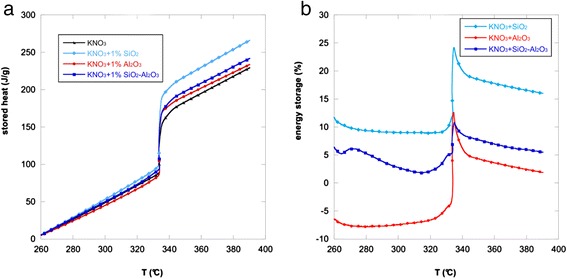
Stored heat and ratio of energy storage versus temperature. Stored heat of KNO_3_ and the nanofluids with 1.0 wt.% of oxide nanoparticles (**a**); ratio of energy storage (total gain %) versus temperature calculated respect to KNO_3_ (**b**)

Figure 4a shows the results obtained for all the nanofluids produced: after the phase change, the curves obtained are all above the one referring to the potassium nitrate. Above the phase change temperature, the curves related to all the nanofluids obtained are shifted with respect to the base salt towards higher stored heat values. In particular, the maximum stored heat of the base salt was 229 J/g, while those of the nanofluids produced were 234, 242, and 266 J/g with the addition of alumina, silica-alumina, and silica nanoparticles, respectively. As it can be noted, the most effective nanoparticles were in descending order: silica, silica-alumina, and alumina.

From the comparison of the heat flow curves and the stored heat curves, it can be noted that after the phase change the hierarchy of the curves changed. This can be explained by analyzing Fig. 1 (insert): the heat flow curves have different shapes and the overall heat stored before and during the phase transformation changes the hierarchy.

The stored heat as the function of temperature allowed the evaluation of the overall heat stored by a material that includes both the sensible heat (before and after the phase change) and the latent heat (during the phase change) stored by each nanofluid between 260 and 390 °C. For this reason the overall behavior is different from the simple heat capacity. In this case in fact not only silica nanoparticles but also the other nanoparticles give an overall stored heat higher than the base salt itself.

The total gain due to the introduction of the nanoparticles was calculated as the ratio between the total stored heat of the nanofluids and the total stored heat of the base salt (Eq. 3):

3 $$ \mathrm{Total}\_\mathrm{gain}\left(\%\right)=\frac{{\displaystyle \underset{T \min }{\overset{T \max }{\int }}{h}_{\mathrm{nanoflluid}}(T)dT}}{{\displaystyle \underset{T \min }{\overset{T \max }{\int }}{h}_{\mathrm{PCM}}(T)dT}}100 $$

Figure 4b shows the total gain (%) versus temperature for all the samples. The peaks indicate the contribution of the latent heats due to the phase change. As it can be seen, the maximum values of the total gain for each nanofluid (at 390 °C) are 16, 6, and 2 % for the nanofluids with 1.0 wt.% of silica, silica-alumina, and alumina nanoparticles, respectively. This means that the phase change materials obtained by mixing potassium nitrate and silica nanoparticles are more effective and can store a higher amount of heat.

### Microstructure of Nanofluids

Since the heat capacity of a material is closely related to its structure, the morphological characterization of the nanofluids prepared was useful in order to characterize the distribution of the nanoparticles in the nanofluid after the calorimetric analysis. A uniform distribution and a homogeneous dispersion of the filler are usually considered responsible of the specific heat increase and corresponding decrease of melting points of the nanofluids. Therefore, SEM microscopy is a powerful tool to understand the factors that are responsible of the thermal properties improvement [20, 27, 49, 50].

**Fig. 5:**
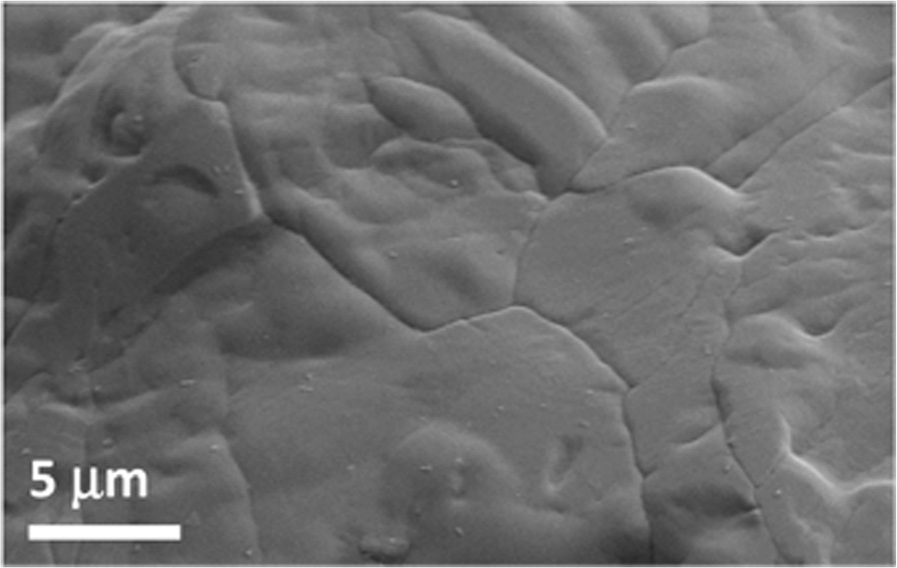
SEM image of KNO_3_. The magnification used is ×10,000

Figure 5 reports a SEM image of the base salt after several melting and solidification cycles. In particular, it represents the morphology of potassium nitrate at ×10,000 that better highlights the grain shape and size.

**Fig. 6:**
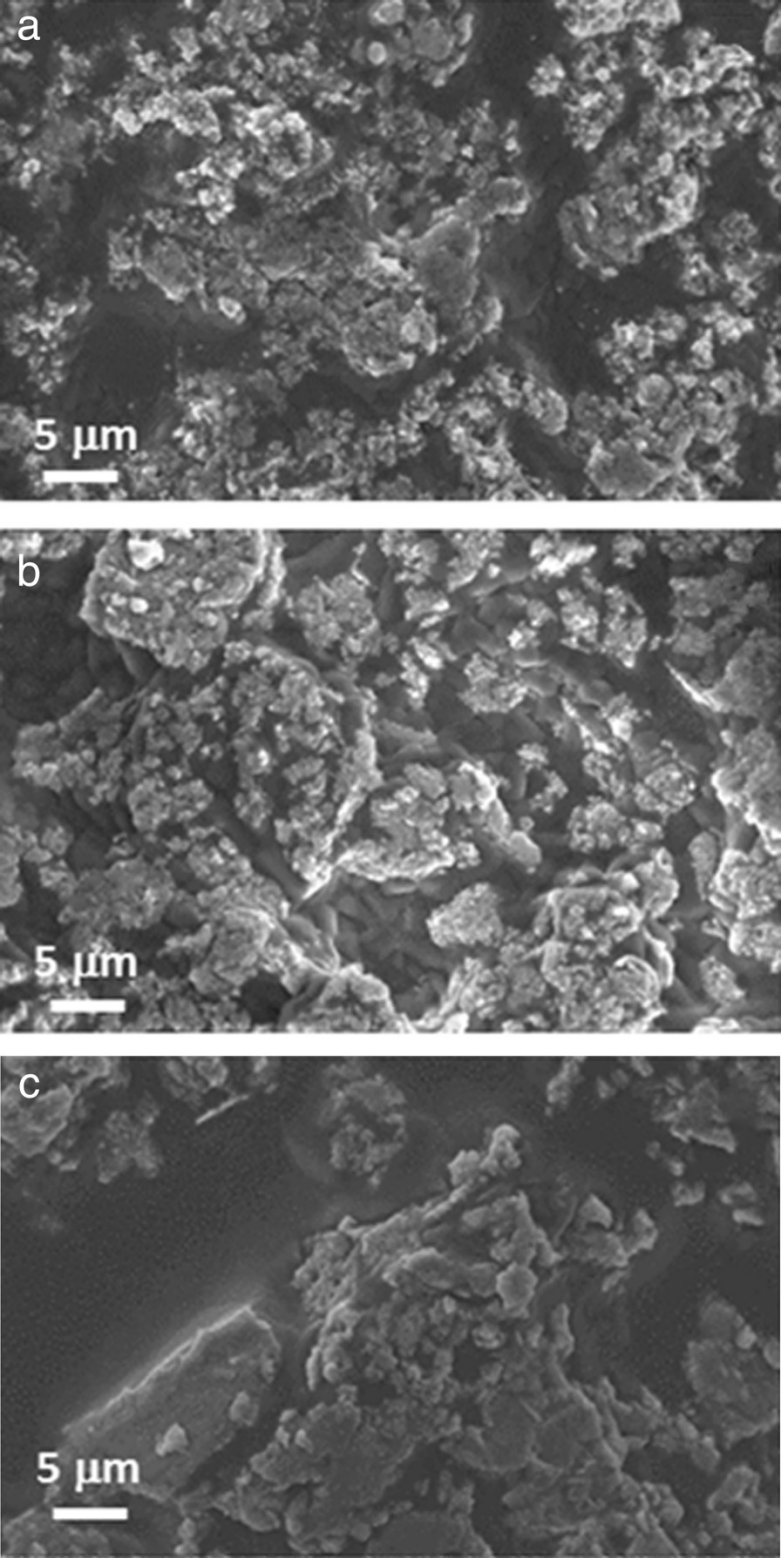
SEM images at ×5,000 magnification. SEM images of the nanofluids based on KNO_3_ with 1.0 wt.% of **a** silica, **b** alumina, and **c** silica-alumina nanoparticles

Figure 6 shows SEM micrographs (at ×5,000 magnification) of the nanofluids produced by adding to potassium nitrate 1.0 wt.% of SiO_2_ (Fig. 6a), Al_2_O_3_ (Fig. 6b), and SiO_2_-Al_2_O_3_ nanoparticles (Fig. 6c). Observing the microstructure of the nanofluids obtained with 1.0 wt.% of silica nanoparticles, it was possible to see a homogenous dispersion of the nanoparticles in the salt with smaller aggregates, while in Fig. 6b (showing the microstructure of the nanofluids with 1.0 wt.% of alumina), it was possible to observe zones characterized by the presence of non-homogenous aggregates of nanoparticles and zones where only pure salt was present. Same observations can be made analyzing the SEM micrographs of the nanofluid obtained with 1.0 wt.% of SiO_2_-Al_2_O_3_ nanoparticles (Fig. 6c). In this case the areas with pure potassium nitrate seemed more extended with respect to the nanofluid with alumina.

**Fig. 7:**
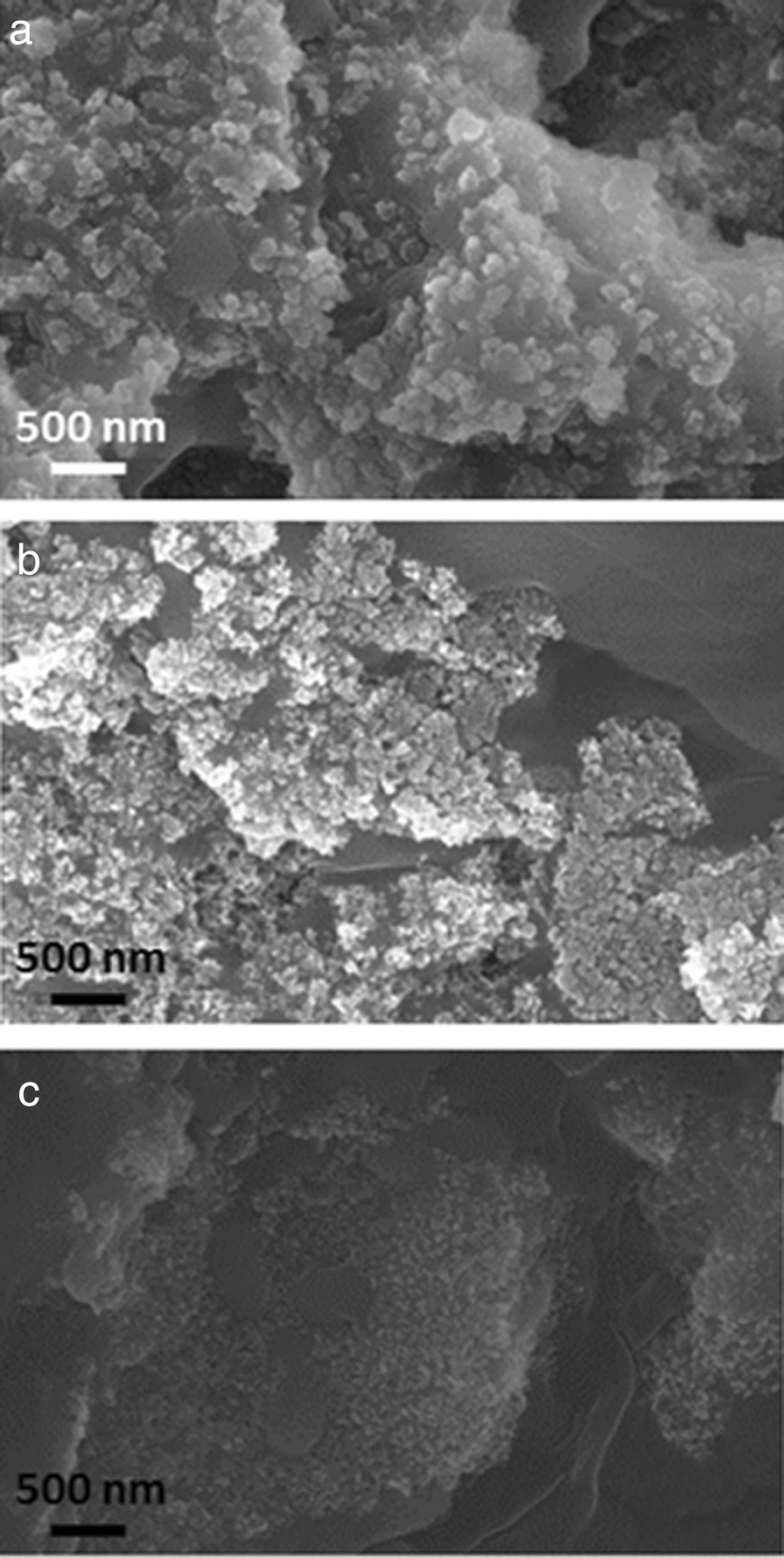
SEM images at ×50,000 magnification. SEM images of the nanofluids based on KNO_3_ with 1.0 wt.% of **a** silica, **b** alumina, and **c** silica-alumina nanoparticles

The SEM micrographs at higher magnification of all the nanofluids produced (×50,000 in Fig. 7) confirm the better distribution of silica nanoparticles into potassium nitrate with a finer dispersion respect to the other nanoparticles used.

Moreover, the comparison of nanofluid micrographs with the base salt (Fig. 5) reveals that the grains in the nanofluids are much smaller than those in the pure salt.

A better dispersion into the salt can be achieved with smaller sized nanoparticles [51, 52].

**Fig. 8:**
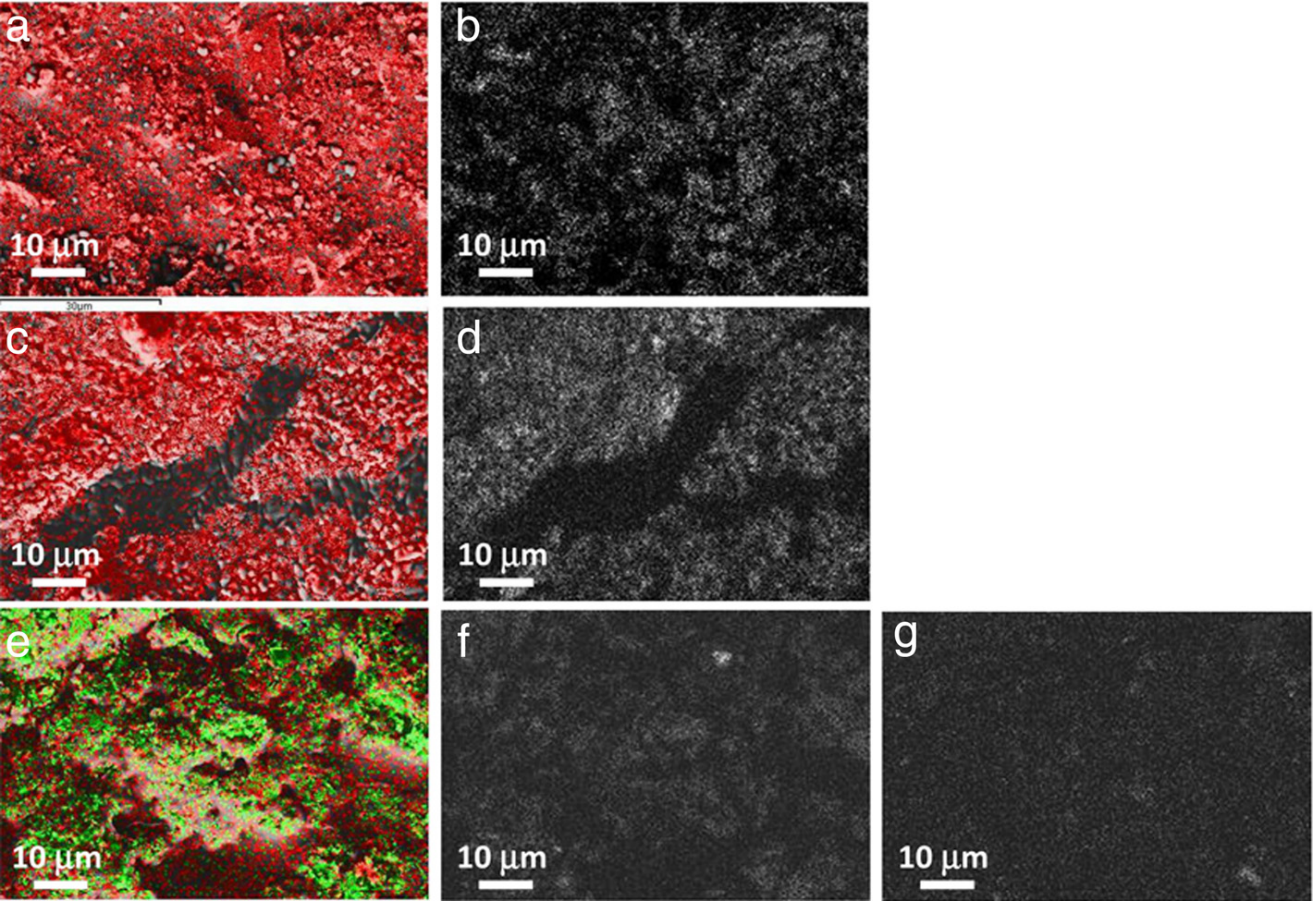
EDX mapping. Nanofluids based on KNO_3_ with 1.0 wt.% of **a**-**b** silica (Si, *red*), **c**-**d** alumina (Al, *red*), **e**-**g** silica-alumina (Si, *green*; Al, *red*) nanoparticles

For a better understanding, energy-dispersive X-ray (EDX) maps of the samples are shown in Fig. 8 where potassium and oxygen are not represented in color in order to highlight the presence of nanoparticles. Figure 8a, b shows the nanofluid with silica nanoparticles: the potassium salt is recognizable in plain and darker regions, while the red color is the presence of silicon that represents silica aggregates. Figure 8c, d shows the nanofluid with alumina nanoparticles: in this case the red color is the aluminum representing alumina aggregates. Finally in Fig. 8e–g the red zones are aluminum while the green zones are silicon. Figure 8f in particular shows the distribution of only silicon and Fig. 8g only aluminum. In this way it is possible to recognize for example a silica aggregate as a light upper zone in Fig. 8f and an alumina aggregate on the lower zone in Fig. 8g both visible in Fig. 8e.

Moreover, EDX was very useful to highlight the distribution of Si and Al in the nanofluids produced with silica-alumina: the green spots (Si) look much more present than the red ones (Al) confirming the presence of more silica (86 %) than alumina in the mixture used.

Elemental mapping reveals that silica nanoparticles are more dispersed in the pure salt while with alumina and silica-alumina large zone of almost pure salt are evident. In any case both SEM and EDX show agglomerated nanoparticles.

In order to explain the enhanced heat capacity of the nanofluids obtained, it is possible at first to refer at the same mechanisms proposed for thermal conductivity: the aggregation of nanoparticles [53–55], the Brownian motion of nanoparticles [56–58], and the formation of a nanolayer [20, 59, 60]. It has been argued that the presence of clusters due to the agglomeration of nanoparticles can increase the thermal conductivity but in our work does not seem to have the same effect on the specific heat. On the other hand, since heat capacity is not a transport property, the Brownian motion of nanoparticles should not be responsible of the observed enhancement. The third proposed mechanism that could lead to an increment of *C*_p_ is the formation of a solid-like nanolayer on the surface of the nanoparticle with higher thermal properties than the bulk liquid. It could contribute to the increase of specific heat of nanofluids [20, 27]. It is possible to arrive at similar conclusions even considering the three modes proposed by Shin to explain the enhanced heat capacity of nanofluids [20]. Based on mode I, the enhancement of specific heat could be explained by the higher heat capacity of nanoparticles than the bulk value of the corresponding materials.

According to mode II the interfacial interaction between the nanoparticles and the surrounding liquid molecules (in particular, the increase in thermal resistance between them due to the high surface area per unit mass of nanoparticles) leads to an increase of heat capacity.

In mode III the liquid molecules on the nanoparticles surface act like a semi-solid layer with higher thermal characteristics than the bulk liquid. The thickness of this layer could be around 2–5 nm as evaluated by molecular dynamics works, and it is directly proportional to the nanoparticle size.

In our work mode I cannot explain the enhancement observed in the nanofluids since the specific heats of the oxide nanoparticles used are lower than the base phase change material. Therefore, mode II and mode III could both be responsible of the anomalous increment of specific heat. In particular, the high surface area of nanoparticles could act as additional thermal storage factor together with the formation of a solid-like nanolayer on the surface of the nanoparticles [61].

Furthermore, while different mechanisms have been proposed as responsible of the thermal conductivity increase and some of them also attributed to the specific heat increase, almost no research has been made to explain the enhancement mechanism for the latent heat of nanofluids. A possible explanation of latent heat enhancement could be the presence of a layer of small agglomerates in the nanofluids in which the nanoparticles would be trapped. The melting of the solid entrapped in these agglomerates would require more energy to occur causing an increase of the latent heat. Anyway, further investigation has still to be done in order to clearly understand and explain the latent heat enhancement in nanofluids.

## Conclusions

Different kinds of nanoparticles (1.0 wt.%) were dispersed in potassium nitrate to obtain high-temperature nanofluids. The nanoparticles added were silica, alumina, and a mixture of silica and alumina (86–14 wt.%). The nanofluids obtained were subjected to calorimetry, and the dispersion of the nanoparticles was analyzed by SEM.

Calorimetric analysis showed that the heat of fusion of the nanofluids obtained with the addition of the nanoparticles to potassium nitrate increased while the onset temperatures were lowered. Moreover, an increase of the specific heat (*C*_p_) ranging from 5 to 9.5 % in the solid phase and of about 6 % in the liquid phase was detected. In particular, the best results were obtained with the addition of SiO_2_ nanoparticles that showed a decrease of about 2–3 °C for the onset temperatures and an increase of 9.5 % for the specific heat. Their addition also provides an increase of 16 % of the total stored heat.

Scanning electron micrographs suggest a higher interaction between the SiO_2_ nanoparticles and potassium nitrate with a better distribution of nanoparticles and absence of zone of pure salt (as EDX also revealed). This work showed that the addition of nanoparticles induces an increase in specific heat of the nanofluids that can be used for thermal storage applications.

In particular, the recorded improvement of about 10 % can led to several important advantages from an engineering perspective. The increased energy density not only implies a reduction of the required amount of the storage medium but also a decrease of the size of thermal energy storage system and a reduction of the number of weldings, insulating material, and material to contain the salt that are needed to build up the overall TES structure, in other words, a larger cost reduction of the TES system. Moreover, as a consequence of these reductions, a decrease in the environmental impact and an increase of sustainability can be achieved.

Adding nanoparticles to potassium nitrate can increase its thermal energy storage capacity. Thus, these new KNO_3_-based nanomaterials can be successfully used as thermal energy storage media in concentrated solar power systems.

## Abbreviations

DSC: differential scanning calorimeter
LHTES: latent heat thermal energy storage
PCM: phase change material
SEM: scanning electron microscopy
TES: thermal energy storage
